# Downregulation of TASK-3 Channel Induces Senescence in Granulosa Cells of Bovine Cystic Ovarian Follicles

**DOI:** 10.3390/ijms251810199

**Published:** 2024-09-23

**Authors:** Chang-Woon Kim, Eun-Jin Kim, Min Seok Woo, Dang Long Cao, Asifiwe Clarisse Cirunduzi, Ji Hyeon Ryu, Il-Keun Kong, Dong Kun Lee, Seong-Geun Hong, Jaehee Han, Dawon Kang

**Affiliations:** 1 Department of Obstetrics and Gynecology, Samsung Changwon Hospital, Sungkyunkwan University School of Medicine, Changwon 51353, Republic of Korea; 2 Department of Physiology, Institute of Medical Sciences, College of Medicine, Gyeongsang National University, Jinju 52727, Republic of Korea; 3 Department of Convergence Medical Science, Gyeongsang National University, Jinju 52727, Republic of Korea; 4 Research Institute for Convergence of Biomedical Science and Technology, Pusan National University Yangsan Hospital, Yangsan 50612, Republic of Korea; 5 Division of Applied Life Science (BK21 Plus), Gyeongsang National University, Jinju 52828, Republic of Korea

**Keywords:** granulosa cell, ovarian cyst, potassium ion, TASK-3 channel

## Abstract

Ovarian cysts are linked to hormone imbalances and altered gene expressions, but the connection between cysts and ion channel expression is understudied. This study explored the role of TWIK-related acid-sensitive K^+^ (TASK) channels in bovine ovarian cyst formation. The ovarian follicles were split into small (5 to 10 mm in diameter) and large (>25 mm in diameter) groups. Among the measured K^+^, Na^+^, and Cl^−^ concentrations in follicular fluid (FF) obtained from small-sized follicles (SFs) and large-sized follicles (LFs), the K^+^ concentration was significantly lower in LFFF. Quantitative PCR, Western blot, and immunocytochemistry data revealed that TASK-3 expression levels significantly decreased by approximately 50% in LFs and granulosa cells obtained from LFs (LFGCs) compared to the corresponding controls. The TASK-3 protein was localized to the plasma membranes of GCs. The diameters of LFGCs were larger than those of SFGCs. The cell swelling response to exposure to a hypotonic solution (200 mOsm/L) was highly reduced in TASK-3-overexpressing cells compared to vector-transfected cells. TASK-3-knockdown cells showed arrested growth. Senescence markers were detected in LFGCs and TASK-3-knockdown cells. These findings suggest that reduced TASK-3 expression in LFs is associated with the inhibition of GC growth, leading to senescence and cyst formation.

## 1. Introduction

Follicular cysts, a common ovarian disorder, contribute to infertility in mammals. These cysts arise when a follicle fails to ovulate at the appropriate time due to various factors, including genetic predisposition, hormonal imbalances, nutrition, aging, gene dysregulation, and stress, leading to cyst enlargement and fluid accumulation [1,2]. Among these factors, disruptions in the hypothalamic–pituitary–ovarian (HPO) axis, causing imbalances in sex hormones, are widely recognized as a significant trigger for cyst formation [3]. However, intraovarian gene alterations and proteins influence ovarian development and folliculogenesis beyond endocrine disturbances, potentially contributing to cyst formation. 

Cystic ovaries exhibit distinct gene and protein expression profiles compared to dominant or healthy follicles [3,4]. The changes in gene expression within ovarian cells are closely linked to the etiology of cystic ovarian conditions [5,6]. In cattle, granulosa cells (GCs) from persistent follicles show specific gene expression patterns associated with changes in follicle size [6,7,8,9]. Ovarian follicles exceeding 25 mm in diameter in cattle are classified as cystic [10], although the precise mechanisms underlying cyst formation are not fully understood. 

GCs are central in regulating follicle growth and maturation, influencing follicle size. GCs secrete various growth factors and signaling molecules that promote oocyte development and produce estrogen, an essential hormone in follicular development. Disruptions of GC function can lead to the formation of cysts, infertility, irregular menstrual cycles, and other reproductive disorders. In cattle with cystic ovaries, abnormal GCs hinder the normal process of folliculogenesis, resulting in the formation of persistent cystic follicles [11,12]. 

Ion channels are pivotal in various cellular processes, including proliferation, apoptosis, senescence, migration, and volume regulation. The movement of water and ions through ion channels at the cell membrane is crucial for regulating ovarian cellular activities, including folliculogenesis [13]. The interplay between large molecules and ions, secreted by oocytes or GCs, precipitates an osmotic pressure gradient that draws water into the antrum [14]. In the formation of the ovarian follicular antrum, cell volume regulation emerges as a cornerstone mechanism. Follicles undergo expansion until osmolality equilibrium is achieved inside and outside the cells. This swelling, in turn, triggers the activation of Cl^−^ or K^+^ channels, which are crucial to the regulatory volume decrease (RVD) and apoptotic volume decrease (AVD) processes [15]. 

While the role of Cl^−^ channels in ovarian folliculogenesis has been explored, there are fewer studies on the involvement of K^+^ channels in ovarian cyst formation. Several K^+^ channels, such as K_ATP_, K_Ca_ (IK, SK, BK), Kv4.2, and two-pore domain potassium (K_2P_) channels, are expressed in the ovary [16,17]. K_2P_ channels regulate osmotic water movement by controlling the resting membrane potential and facilitating K^+^ efflux in various cells, including mouse zygotes [18]. In the mammalian ovary and female germ cells, the TWIK-related acid-sensitive K^+^ channels (TASKs: TASK-1, TASK-3, and TASK-5), belonging to the K_2P_ channel family, are expressed [17,19,20]. In addition, TASK-2, another member of the K_2P_ channel family, has been associated with apoptosis through its role in volume regulation [21]. In breast cancer cells, the downregulation of TASK-3 enhances cell migration and invasion [22], while also inducing cellular senescence and inhibiting growth [23]. Given their role in regulating cell volume and growth, TASK channels are likely to influence the formation of ovarian cysts.

This study investigates the potential role of TASK channels in forming cystic follicles in bovine ovaries. Specifically, we compare ion concentration and TASK channel expression levels in follicular fluid (FF) and GCs obtained from small-sized (5 to 10 mm in diameter) and large-sized (>25 mm) follicles (SFs and LFs) in Korean cattle.

## 2. Results

### 2.1. Low K^+^ Concentration in Follicular Fluid Obtained from Large-Sized Follicles

**Figure 1:**
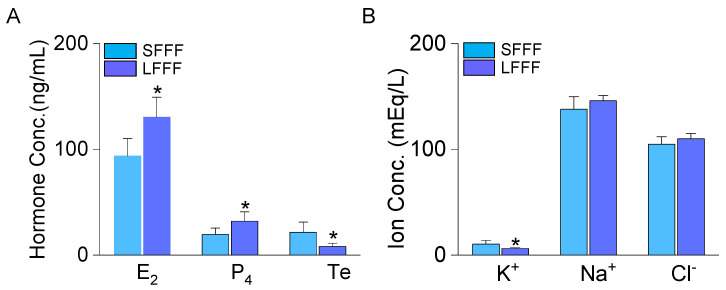
Hormone and ion concentrations in bovine ovarian follicular fluid obtained from small-sized and large-sized follicles. (**A**) The concentrations of 17β-estradiol (E_2_), progesterone (P_4_), and testosterone (Te). (**B**) The concentrations of K^+^, Na^+^, and Cl^−^. Each bar represents the mean ± SD of five different samples. * Significant difference from the corresponding control value (*p* < 0.05). SFFF and LFFF denote follicular fluid obtained from small-sized (5 to 10 mm) and large-sized (>25 mm) follicles, respectively.

Ovarian follicles were categorized based on their diameter, with sizes ranging from 5 to 10 mm (5~10 mm) or exceeding 25 mm (>25 mm). The smaller follicles were referred to as small-sized follicles (SFs), while the larger ones were referred to as large-sized follicles (LFs). Comparing the follicular fluid (FF) obtained from LFs (LFFF) to that obtained from SFs (SFFF), significantly higher concentrations of estrogen (17β-estradiol, E_2_) and progesterone (P_4_) were observed in LFFF. In contrast, the concentration of testosterone (Te) decreased by approximately 63% in LFFF (Figure 1A; *p* < 0.05). Analysis of the concentrations of K^+^, Na^+^, and Cl^−^ in SFFF revealed values of 10.4 ± 3.5 mEq/L, 138.0 ± 11.9 mEq/L, and 104.9 ± 7.0 mEq/L, respectively. The K^+^ concentration was significantly lower in LFFF (6.2 ± 0.8 mEq/L, Figure 1B; *p* < 0.05). There were no significant differences in Na^+^ and Cl^−^ concentrations between SFFF and LFFF (*p* > 0.05). These results suggest that the regular movement of K^+^ is disrupted in the LFs. 

### 2.2. Downregulation of TASK Expression Levels in GCs Obtained from LFs

**Figure 2:**
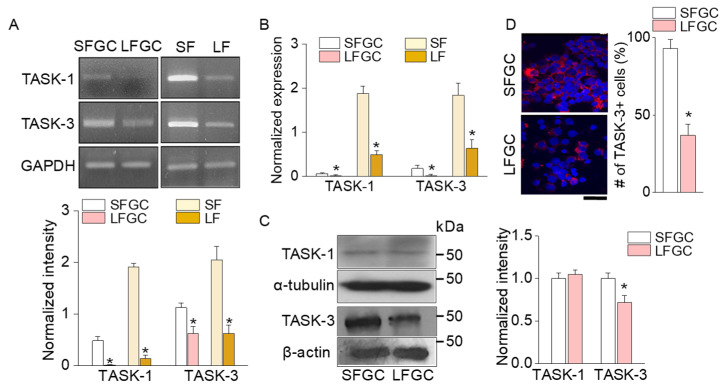
Downregulation of TASK-3 expression levels in bovine ovarian follicles and granulosa cells. (**A**) TASK-1 and TASK-3 mRNA expression in follicles (SFs and LFs) and GCs (SFGC and LFGC). TASK-1 (287 bp) and TASK-3 (433 bp). (**B**) Real-time PCR data for TASK-1 and TASK-3 in follicles and GCs. The expression levels were normalized to GAPDH. (**C**) Western blot analysis of TASK-1 and TASK-3 in SFGC and LFGC. The protein expression levels were normalized to α-tubulin. (**D**) TASK-3 immunofluorescence intensity in GCs. The GCs isolated from each ovary were cultured on a glass coverslip and subjected to immunostaining to detect the expression of TASK-3 within the cells. TASK-3 was immunostained with an anti-TASK-3 antibody and cyanine (Cy3)-conjugated anti-rabbit IgG (red). 4′,6′-diamidino-2-phenylindole (DAPI) was used for nuclear staining (blue). Each bar represents the mean ± SD of four different samples. * Significant difference from the corresponding control value (*p* < 0.05). Scale bar, 50 μm.

The TASK channels, known as one of the K^+^ transport systems in bovine ovarian follicles, were investigated in this study. Our investigation was limited to TASK-1 and TASK-3, as TASK-5 does not exhibit functional expression in the recombinant system, and the formation of heterodimers between TASK-5 and TASK-1 or TASK-3 remains unverified [24]. Semi-quantitative PCR data showed a significant decrease in mRNA levels of TASK-1 and TASK-3 in both LFs and GCs obtained from LFs (LFGC) when compared to SFs and SFGC (Figure 2A, *p* < 0.05, n = 4). Real-time PCR data further confirmed a significant reduction in the mRNA expression levels of TASK-1 and TASK-3 in LFs and LFGC compared to SFs and SFGC (Figure 2B; *p* < 0.05, n = 4). In addition to mRNA levels, the protein level of TASK-3 was also found to be decreased in LFGC (Figure 2C; *p* < 0.05, n = 5). However, no significant change was observed in the protein level of TASK-1 between SFGC and LFGC (Figure 2C; *p* > 0.05, n = 4). Immunofluorescence analysis revealed that the intensity and number of TASK-3-positive cells are lower in LFGC than in SFGC (Figure 2D). The changes observed in both the mRNA and protein levels of TASK-3 channels in LFGC were subsequently subjected to further analysis through immunostaining. 

### 2.3. Localization of TASK-3 in Ovarian GCs 

**Figure 3:**
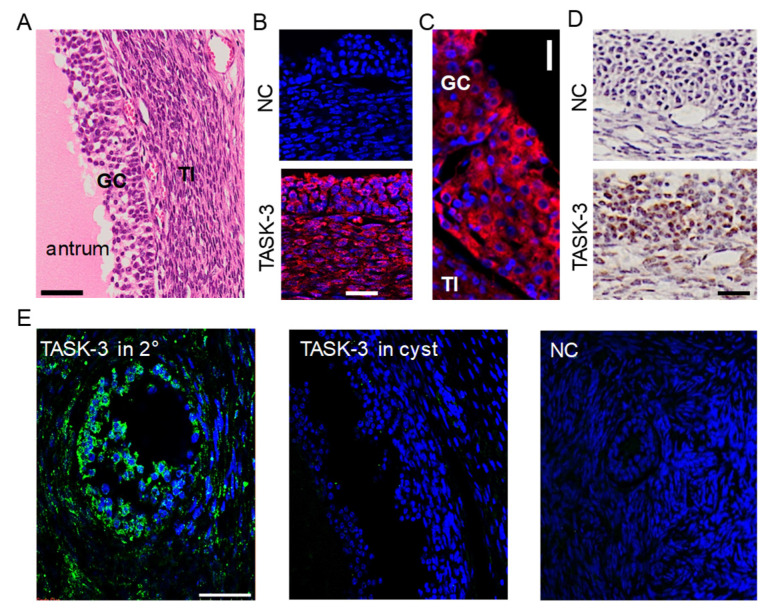
Expression of TASK-3 in granulosa cells. (**A**) H&E staining of a bovine ovary tissue section. GC and TI represent granulosa cells and theca interna, respectively. (**B**) Expression of TASK-3 in both GC and TI. Fluorescent images labeled with anti-TASK-3 antibody and cyanine (Cy3)-conjugated anti-rabbit IgG (orange/red). 4′,6′-diamidino-2-phenylindole (DAPI) was used for nuclear staining (blue). (**C**) An enlarged immunofluorescence image showing the TASK-3 channel (red) and DAPI staining for nuclei (blue). Scale bar, 10 μm. (**D**) TASK-3 expression in GC detected by DAB staining. (**E**) The immunofluorescence intensity of TASK-3 detected in follicles of different sizes. Green and blue indicate TASK-3 expression and nucleus stained with Alexa Fluor 488 and DAPI, respectively. The 2° and cyst labels correspond to secondary and cystic follicles, respectively. NC represents negative control, omitting anti-TASK-3 antibody. Scale bars, 50 μm.

Two different immunohistochemical methods, fluorescence and 3, 3′-diaminobenzidine (DAB) staining, were employed to examine the expression pattern of TASK-3 in the ovary. The ovary, excluding follicles larger than 25 mm, was subjected to hematoxylin and eosin (H&E) staining and histological examination, revealing the presence of antrum, GC, and theca interna (TI) (Figure 3A). Immunofluorescence staining showed TASK-3 expression in both GCs and TI as shown in Figure 3B, while DAB staining predominantly highlighted TASK-3 expression in GCs (Figure 3D), indicating that the GC is the primary site of TASK-3 expression in the ovary, with the possibility of its presence in TI. An enlarged immunofluorescence image showed that TASK-3 is localized on the GC’s plasma membrane and cytoplasm (Figure 3C). As shown in Figure 3E, fluorescence-based analysis revealed higher levels of TASK-3 expression in small-sized follicles compared to large-sized (cystic) follicles. 

### 2.4. Senescence Signals Increased in LFGC

**Figure 4:**
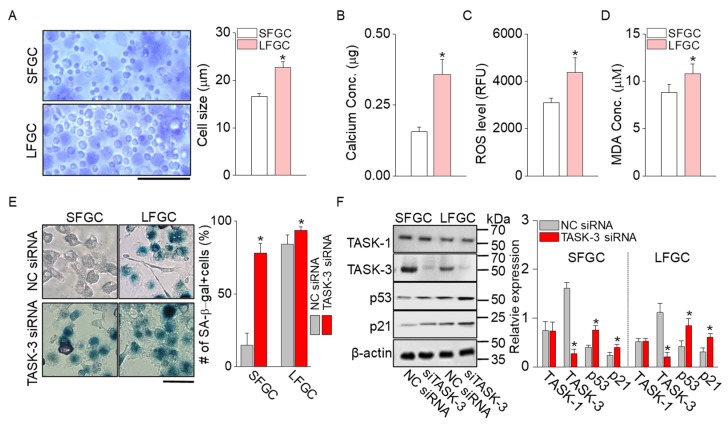
Upregulation of senescence signals in LFGC. (**A**) Trypan blue-stained cells. The size of the cells was measured with a software ruler. (**B**) Calcium concentration in GCs. (**C**) ROS levels in GCs. RFU represents the relative fluorescence unit. (**D**) MDA concentration in GCs. The label referenced in (**D**) was common to (**B**,**C**). (**E**) Representative images stained with SA-β-gal. Images depict cells stained with SA-β-gal, which appear in dark cyan when stained. (**F**) Detection of p53 and p21 proteins. The expression levels were normalized to β-actin. Each bar represents the mean ± SD of four to six samples. * Significant difference from the corresponding control value (*p* < 0.05). Scale bars, 100 μm.

Senescence markers such as cell volume, concentrations of calcium, reactive oxygen species (ROS), malondialdehyde (MDA), and SA-β-galactosidase signals were analyzed in GCs. The diameter of LFGC (22.7 ± 1.2 µm, n = 288) was larger than that of SFGC (16.6 ± 0.7 µm, n = 257) (Figure 4A), indicating that low expression of TASK-3 may induce cellular senescence and hinder water efflux. The majority (approximately 90%) of SFGC had a size of 15.38 µm. Measurement of calcium, ROS, and MDA concentrations in GCs revealed significantly higher levels in LFGC compared to SFGC (Figure 4B–D; *p* < 0.05, n = 6). Furthermore, the number of SA-β-galactosidase-positive (SA-β-gal+) cells was significantly higher in LFGC compared to SFGC (Figure 4E). In both SFGC and LFGC groups, TASK-3 knockdown increased the number of SA-β-gal+ cells. In addition, LFGC exhibited elevated expression levels of p53 and p21 compared to SFGC. Knockdown of TASK-3 in both groups also resulted in heightened expression of p53 and p21 (Figure 4F; *p* < 0.05, n = 4). As shown in Figure 4F, while the TASK-3 protein expression level decreased in the TASK-3 knockdown cells, TASK-1 protein expression remained unchanged. 

### 2.5. Hypotonic-Induced Swelling Reduced by TASK-3 Activation 

**Figure 5:**
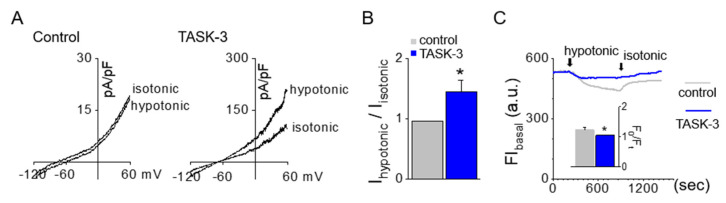
Volume regulation by the TASK-3 channel. (**A**) Activation of the TASK-3 channel by hypotonic solution. The whole-cell current of TASK-3 was responsive to the tonicity shift in the extracellular solution from 300 mOsm to 200 mOsm. (**B**) Summary of changes in TASK-3 currents upon applying a hypotonic solution. (**C**) Volume changes in response to a hypotonic solution. The volume changes were monitored by examining the time course of relative fluorescence. Bar graphs showed fluorescence intensity after a 6 min exposure to the hypotonic solution. Each bar represents the mean ± SD of four different samples. * Significant difference from the corresponding control value (*p* < 0.05). FI represents fluorescence intensity. F_0_ signifies the fluorescence intensity in an isotonic solution at time *=* 0. The ratio F_0_/F_t_ correlates with cell volume. Control refers to cells transfected with the pcDNA3.1 vector. The a.u. stands for an arbitrary unit. Isotonic and hypotonic solutions have tonicities of 300 mOsm and 200 mOsm, respectively.

To determine the role of TASK-3 in volume regulation, CHO cells transfected with TASK-3 were exposed to a hypotonic solution possessing an osmolarity of 200 mOsm. Introducing this hypotonic solution resulted in a boost of approximately 30% in TASK-3 currents (Figure 5A,B, n = 4). The cell’s response to the hypotonic solution was monitored by measuring the time course of basal fluorescence intensity (FI_basal_) and F_0_/F_t_ (proportional to cell volume) in cells loaded with calcein. A decrease in FI corresponds to an increase in cell volume. As control cells encounter a hypotonic solution, they rapidly swell, decreasing fluorescence intensity. However, cells overexpressing TASK-3 exhibited a negligible shift in fluorescence intensity compared to those transfected with the control vector. The overexpression of TASK-3 significantly curtails the cell’s swelling when subjected to the hypotonic solution (Figure 5C, *p* < 0.05, n = 8), suggesting that cells overexpressing TASK-3 can recover from swelling to basal levels after being exposed to these conditions. 

## 3. Discussion

Ovarian follicles larger than 25 mm in diameter are classified as cystic in cattle [10]. In this study, follicles exceeding 25 mm were characterized as follicular cysts based on histological features and hormonal profiles, specifically an E_2_/P_4_ ratio greater than 1 and low testosterone levels. Follicular cysts, typically fluid-filled sacs on the ovarian surface, develop when a follicle fails to rupture and release its oocyte due to hormonal dysregulation. While our study focused on gene expression changes, the effect of hormones on follicular cyst development cannot be dismissed, given that hormones such as E_2_, P_4_, and testosterone are known to regulate ion channel activity, especially K^+^ channels [25,26,27]. The reduced K^+^ concentration ([K^+^]) observed in the LFFF may be linked to the suppression of K^+^ channel expression or activity in the GCs. Although E_2_ is known to inhibit TASK-1 and TASK-2 [28,29], the regulation of TASK-3 remains unexplored. 

We analyzed the expression levels of TASK-1 and TASK-3 in follicles and GCs. While both TASK-1 and TASK-3 were expressed at similar levels in follicles, TASK-3 showed significantly higher expression in GCs, suggesting a preferential role in these cells. This may indicate that TASK-1 is more prominently expressed in theca cells, oocytes, or histiocytes that may have been collected during follicle harvesting. The lower [K^+^] in LFFF compared to SFFF provided insight into potential alterations in K^+^ channel expression. Consistent with previous findings, [K^+^] decreased as follicle size increased [30]. However, no significant changes were observed in Na^+^ or Cl^−^ concentrations between small and large follicles, ruling out dilution as the cause of decreased [K^+^]. This isolated reduction in [K^+^] likely stems from dysregulated K^+^ channels or transporters, essential for maintaining resting membrane potential and other cellular functions [31]. In normal conditions, extracellular [K^+^] is tightly regulated, typically ranging from 3.5 to 5.0 mmol/L, and disruptions in [K^+^] can impact processes such as cell growth, apoptosis, and volume regulation [32,33]. A relatively high [K^+^] is sustained in FF. Previous studies suggest that [K^+^] levels in FF are typically similar to those in body fluids. However, FF from ovaries obtained from slaughterhouses has shown elevated [K^+^] levels compared to body fluid [34], possibly due to impaired K^+^ transport or altered protein expression in these ovaries. Further investigation is needed to determine the cause of this disparity. The ovaries in this study were sourced from slaughterhouses and exhibited elevated [K^+^] levels, which warrants further exploration.

Dysfunctional K^+^ channels in the reproductive system can impair the HPO axis, reducing fertility [35]. Ovarian K^+^ channels play crucial roles in the synthesis and secretion of E_2_ and P_4_ [16,36], which are vital for follicular development. GCs release hormones, with a substantial portion of GC-produced estrogen entering the FF. Estrogen receptors alpha and beta (ERα and ERβ) act as hormone-responsive transcription factors regulating target gene expression, including TASK-3. Using the transcription element search system (TESS), we identified three predictive estrogen response elements in the TASK-3 promoter region. E_2_ treatment upregulated TASK-3 expression in GCs. However, ERα and ERβ expression was lower in LFGCs than in SFGCs, which may limit E_2_’s influence on TASK-3 expression. This reduced expression of ERs in LFGCs may explain the decreased TASK-3 expression observed in these cells. The reduction in TASK-3 expression in GCs likely impairs K^+^ efflux, disrupts RVD, leads to membrane depolarization, and triggers cellular senescence. TASK-3 activation in GCs regulates osmotic balance by driving water efflux, which is essential for maintaining the FF volume [14,37]. AQP4 may collaborate with TASK-3 in facilitating water and ion transport in GCs [38], but its reduced levels in LFGCs [39] suggest limitations in this transport process. 

Cellular senescence is a state of permanent growth arrest in which cells cannot proliferate despite optimal conditions [40]. The mechanism of ovarian senescence is similar between cattle and humans, involving disruption of antioxidant signaling in early-stage oocytes and GCs [41]. GCs show downregulation of antioxidative genes in humans, contributing to ovarian dysfunction [41]. Silencing TASK-3 channels in melanoma cells leads to mitochondrial depolarization and apoptosis, highlighting TASK-3′s role in protecting cells from damage [42,43]. TASK-3 is localized in the inner mitochondrial membrane, interacting with the respiratory chain and regulating mitochondrial function [44,45,46]. Given its role in cellular bioenergetics, TASK-3 may also influence cellular senescence. Senescent cells exhibit distinct traits such as cellular enlargement, increased SA-β-gal activity, upregulated cell cycle regulators (p53 and p21), and an amplified senescence-associated secretory phenotype (SASP) [40,47]. Lower TASK-3 expression in LFGCs correlates with increased cell volume, higher SA-β-gal activity, and elevated p53 and p21 expression, suggesting that reduced TASK-3 expression may promote GC senescence. This is further supported by increased ROS and Ca^2+^ levels in LFGCs, both linked to senescence [48,49]. The oncogenic potential of TASK-3 has been documented in several studies [22,50]. While TASK-3 has been implicated in cancer progression, its role in other pathological processes, such as cyst formation, may involve different mechanisms. Senescence, although protective against malignancy, can disrupt tissue homeostasis and contribute to disease processes like cyst formation through the SASP. The complex interplay between TASK-3, cellular senescence, K^+^ channel activity, and tissue homeostasis in cyst formation deserves further investigation.

This study has several limitations. First, the ovaries used for cell isolation and gene analysis were sourced from a slaughterhouse, limiting our control over biological, genetic, and environmental factors that could affect ovarian cyst development and TASK-3 expression. Determining the exact age of the cows was also challenging, but they were estimated to be between 18 and 26 months, within their reproductive years. Only healthy cattle are typically sent to slaughterhouses, though approximately 20% of cattle develop follicular cysts [51]. In South Korea, strict regulations prohibit antibiotics and hormones before slaughter, and all cattle intended for food are tested for these substances. Hormones are not used to treat cysts before slaughter. 

Additionally, while TASK-3 mRNA and protein levels differed between SFs and LFs, only TASK-1 mRNA showed variation. Generally, protein levels are expected to align with mRNA levels across tissues [52]. However, this is not always the case due to factors like post-transcriptional regulation, translation efficiency, and protein degradation [53]. Given the changes observed at both mRNA and protein levels, TASK-3 appears to have a stronger association with ovarian cysts than TASK-1, which is why we focused on it. However, the role of TASK-1 warrants further exploration in future studies. Immunostaining could also not accurately represent TASK-3 expression levels in SFs and LFs, likely due to challenges in properly fixing entire cystic follicles.

It is plausible that decreased expression of TASK-3 channels in GCs of cystic follicles contributes to reduced [K^+^]. Since TASK-3 channels are essential for regulating K^+^ transport across the cell membrane, reduced expression could lead to decreased K^+^ efflux, resulting in lower K^+^ levels in the follicular fluid. This effect may be compounded by hormonal imbalances and circulatory changes associated with large follicular cysts. As the cystic follicle enlarges, compromised blood flow and altered hormonal regulation could further suppress TASK-3 expression, exacerbating the reduction in [K^+^]. These factors likely work together to explain the observed [K^+^] changes in cystic follicles.

## 4. Materials and Methods

### 4.1. Sample Preparation

All reagents were obtained from Sigma (St. Louis, MO, USA), unless specified otherwise. Bovine ovaries were obtained from a slaughterhouse and delivered to the laboratory within 2 h, preserved at 35~39 °C in phosphate-buffered saline (PBS) containing 100 IU/mL of penicillin and 0.1 mg/mL streptomycin. Ovarian follicles were categorized based on their size: small (5 to 10 mm in diameter) and large (>25 mm in diameter). Ovaries with follicles exceeding 25 mm in diameter without a corpus luteum in either the right or left ovary were identified as follicular cystic ovaries. The large follicles were isolated from follicular cystic ovaries. Follicles were prepared by cutting the perifollicular region with a razor blade. Experimental methods used in this study were partially modified from the procedures performed in previous studies [54,55]. The 80 ovaries with large-sized follicles (LFs) and 120 ovaries with only small-sized follicles (SFs) were used in this study. Follicular fluid (FF) and granulosa cells (GCs) obtained from small-sized follicles (5 to 10 mm) of 5~10 ovaries were pooled and used for the experiments as a sample. 

### 4.2. Isolation of Follicular Fluid and Granulosa Cells 

FF and GCs were isolated as previously described [39]. FF was carefully aspirated in follicles with a 10 mL syringe fitted with an 18- or 23-gauge needle. The fluid was centrifuged at 1750× *g* for 10 min, and the supernatant and resultant pellets were used as FF and GCs, respectively. To analyze their number and size, GCs were stained with trypan blue solution (0.4%, Thermo Fisher Scientific, Rockford, IL, USA). GCs suspended in PBS were incubated with an equal volume of trypan blue reagent for 10 min at room temperature. The cells were washed twice with PBS and observed under a BX-51 microscope (Olympus, Tokyo, Japan).

### 4.3. Measurement of 17β-Estradiol (E_2_), Progesterone (P_4_), and Testosterone

Hormone concentration was measured in detail in references [54,55]. E_2_, P_4_, and testosterone levels in the FF were ascertained using the Dissociation Enhanced Lanthanide Fluorescence Immunoassay (DELFIA) system (PerkinElmer Life and Analytical Sciences, Wallac Oy, Turku, Finland). Following the manufacturer’s guidelines, each strip was first washed with DELFIA Platewash. Subsequently, 25 µL of E_2_ standards or FF samples were dispensed into the strip wells. This was followed by adding 100 µL of the diluted E_2_ antiserum solution to each well, and the arrangement was then incubated for 30 min at room temperature with a slow shake. After this incubation, a solution of diluted E_2_ (50:1) in an amount of 100 µL was added to each well. The plate was then incubated for 2 h at room temperature with slow shaking. After this incubation, each strip underwent six washes before an enhancement solution of 200 µL was introduced into every well. A subsequent gentle shake of the plate for 5 min was carried out. The fluorescence levels were recorded using a time-resolved fluorometer (Wallac 1420 VICTOR2^TM^, PerkinElmer, Waltham, MA, USA) within 1 h of this final shaking procedure. Both standards and FF samples were assessed in duplicate. To evaluate P_4_ and testosterone, the same protocol for E_2_ was applied, except for a dilution stage that was conducted before antiserum addition. 

### 4.4. Measurement of Ion Concentrations

Potassium (K^+^), sodium (Na^+^), and chloride (Cl^−^) ion concentrations in the FF were determined with an automatic analyzer (DRI-CHEM 3500i, FUJIFILM, Tokyo, Japan) in line with the manufacturer’s guidelines. For quantitative analysis of the electrolytes in the FF, the potentiometric difference was assessed between two electrodes; one electrode was in contact with a reference liquid holding a known electrolyte concentration, while the other interfaced with the samples. 

### 4.5. Reverse Transcriptase (RT)-Polymerase Chain Reaction (PCR) and Real-Time PCR 

As previously described [54,55], RT-PCR procedures were performed utilizing specific primer pairs, as detailed in Table 1. Bovine follicles and GCs were the source of total RNA, which was isolated using the Trizol reagent (Invitrogen, Carlsbad, CA, USA) following the manufacturer’s guidelines. The resultant total RNA (3 μg) underwent first-strand cDNA synthesis using oligo dT (SuperScript First-Strand Synthesis System, Invitrogen), which then served as a template for PCR amplification employing *Taq* polymerase (Takara Bio Inc., Otsu, Shiga, Japan). The synthesized first-strand cDNA’s concentration was determined with a spectrophotometer (NanoDrop^®^ ND-1000, NanoDrop Technologies, Wilmington, DE, USA) and subsequently employed as the PCR amplification template. The amplification process consisted of an initial denaturation at 94 °C for 5 min followed by 28 or 30 cycles at 94 °C for 20 s, 55 °C for 20 s, 72 °C for 20 s, and a 10 min extension at 72 °C. Amplified products were run on 1.5% agarose gels with ethidium bromide. Resultant bands were extracted and underwent direct sequencing using an ABI PRISM^®^ 3100-Avant Genetic Analyzer (Applied Biosystems, Foster City, CA, USA). 

For real-time PCR, a combination of the Topreal^TM^ qPCR 2X PreMIX kit (Enzynomics, Daejeon, Republic of Korea), sapphire microplate, 96 wells, (Greiner bio-one, Kremsmünster, Austria), and the Light Cycler^®^ 480 II System (Roche, Rotkreuz, Switzerland) was utilized. The PCR parameters included a denaturing cycle (95 °C for 10 min), 45 cycles of PCR (95 °C for 7 s, 56 °C for 7 s, and 72 °C for 10 s), a melting cycle (95 °C for 0 s and 65 °C for 60 s). Relative mRNA levels were determined using the 2^−ΔΔCT^ method [56]. In RT-PCR and real-time PCR analyses, target gene expression was standardized against glyceraldehyde-3-phosphate dehydrogenase (*GAPDH*) levels. 

### 4.6. Western Blot Analysis

Western blotting was conducted based on established protocols [54,55]. GCs underwent homogenization in RIPA lysis buffer (Cell Signaling Technology, Danvers, MA, USA) supplemented with 20 mM Tris-HCl (pH 7.5), 150 mM NaCl/1 mM Na_2_EDTA, 1 mM EGTA, 1% NP-40, 1% sodium deoxycholate, 2.5 mM sodium pyrophosphate, 1 mM β-glycerophosphate, 1 mM Na_3_VO_4_, and 1 μg/mL leupeptin). After incubation at 4 °C for 30 min, the samples were centrifuged at 13,000 rpm (16,609× *g*, Micro 17TR, Hanil, Republic of Korea) for 30 min at 4 °C. The protein content of the supernatants was quantified using the Bradford protein assay (Bio-Rad, Hercules, CA, USA). The proteins (50–100 μg/lane) were then subjected to 10% SDS–polyacrylamide gels, followed by transfer to polyvinylidene fluoride (PVDF) membranes (0.45 μm, Millipore, Bedford, MA, USA) in TBS buffer solution containing 25 mM Tris-base, 190 mM glycine, and 20% methanol. Ponceau S staining verified effective transfer, and the destained blots were blocked. Primary antibodies specific to TASK-1 (1:1000 dilution, Alomone Labs, Jerusalem, Israel), TASK-3 (1:1000 dilution), p53 (1:200 dilution, Santa Cruz Biotechnology, Inc., Dallas, TX, USA), p21 (1:200 dilution, Santa Cruz Biotechnology), α-tubulin (1:5000 dilution), and β-actin (1:5000 dilution) were applied and left overnight at 4 °C. Afterward, blots were incubated with appropriate horseradish peroxidase (HRP)-conjugated secondary antibodies (1:3000; Assay Designs, Ann Arbor, MI, USA) at room temperature for 1 h, and protein bands were visualized using enhanced chemiluminescence (ECL Plus kit; ELPIS, Daejeon, Republic of Korea).

### 4.7. Hematoxylin–Eosin (H&E) Staining 

H&E staining of ovaries was carried out based on the methods detailed in previous studies [54,55]. After a PBS (0.1 M, pH 7.0) wash, the ovaries were fixed in a 4% (*w*/*v*) paraformaldehyde solution, creating 4 μm thick paraffin-embedded sections. For histological assessment of follicles, H&E staining was applied. The sections were laid on gelatin-coated slides and allowed to air dry before deparaffinization. After rinsing with tap water, they were submerged in hematoxylin for 5 min and then validated for thorough staining. A 3 min eosin stain followed. After that, the sections were progressively dehydrated using a graded series of alcohols (70% to 100% ethanol, 3 min each), cleared in xylene, and mounted. Images of the stained sections were captured with a BX-51 microscope (Olympus, Tokyo, Japan) equipped with a high-resolution Camedia C-7070 camera (Olympus). For each sample, five sections underwent evaluation. 

### 4.8. Immunostaining 

Deparaffinized tissue sections were washed in PBS, treated with 0.3% H_2_O_2_ for 30 min, and then rinsed in PBS. To reduce non-specific IgG binding, sections were blocked using 1.5% normal goat serum in PBS at room temperature for 30 min. The sections were then incubated overnight at 4 °C in a humidified chamber with an anti-TASK-3 primary antibody (1:200 dilution). Following another PBS wash, sections were treated either with cyanine 3 (cy3) anti-rabbit secondary antibody (1:400 dilution, Abcam, Cambridge, UK), Alexa Fluor 488 anti-rabbit secondary antibody (1:400 dilution, Invitrogen), or biotin-conjugated secondary antibody (1:200 dilution) diluted in 1.5% normal blocking serum at room temperature for 1 h, followed by three PBS washes. Immunofluorescence staining was counterstained with 4′,6-diamidino-2-phenylindole (DAPI) for visualization. For the 3,3′-diaminobenzidine tetrahydrochloride (DAB) staining, sections exposed to biotin-conjugated secondary antibody were further treated with an avidin-biotin-peroxidase complex (ABC Elite kit; Vector Laboratories, Burlingame, CA, USA) for 1 h at room temperature. These samples were washed with PBS and stained using DAB solution containing 0.03% H_2_O_2_ for 3 min. Hematoxylin was used for counterstaining. Images were captured using a confocal laser scanning microscope (Olympus) for fluorescence and a BX-51 microscope (Olympus) for DAB images. 

For immunocytochemistry, the isolated GCs were cultured on a cover glass for 24 h. The cells were permeabilized with 0.2% Triton X-100 for 10 min at room temperature and then washed thrice in PBS. Blocking was performed using a buffer containing 2% normal goat serum in 0.1 M PBS for 1 h at room temperature. Cells were then incubated overnight at 4 °C with rabbit polyclonal anti-TASK-3 primary antibody (1:100 dilution, Alomone Labs), followed by three additional PBS washes. Cells were next treated with Cy3-conjugated anti-rabbit IgG secondary antibody (1:100 dilution; Invitrogen) for 1 h in the dark, followed by another three washes. Nuclear staining was carried out using DAPI at a concentration of 0.1 μg/mL. Finally, cells were wet mounted using a Permount mounting medium (Fisher Chemical, Geel, Belgium) and observed under an Olympus confocal laser scanning microscope.

### 4.9. Measurement of Free Radical Activity and Calcium and Malondialdehyde (MDA) Concentrations in GCs

The free radical activity and calcium and MDA concentrations were determined following the methodologies described in a previous study [57]. The Oxiselect^TM^ In Vitro ROS/RNS assay kit (Cell Biolabs, San Diego, CA, USA) was utilized to evaluate free radical activity in cell lysates. The Calcium Detection Assay kit (Abcam) was used to measure calcium concentration. MDA concentration was gauged using the OxiSelect™ TBARS assay kit (STA-330; Cell Biolabs). 

### 4.10. Cellular Senescence Assay 

Cellular senescence was evaluated using methods previously described [58] and following the manufacturer’s instructions (BioVision Inc., Milpitas, CA, USA). Briefly, bovine GCs at a 2 × 10^4^ cells/mL density were plated in a 24-well plate and incubated for 24 h at 37 °C and 5% CO_2_. After this incubation, the cells were rinsed twice with PBS and fixed with a fixative solution for 15 min at room temperature. After fixation, the cells were washed thrice with PBS. A staining solution mixture (comprising 470 µL of staining solution, 5 µL of staining supplement, and 25 µL of 20 mg/mL X-gal in DMF) was then added to each well, and the plate was incubated for an additional 48 h at 37 °C. Images were captured from five distinct areas per dish using a microscope (Axiovert 40C, Zeiss, Jena, Germany), and cells were counted to determine the average stained cell number. The percentage of SA-β-gal-positive cells in each sample was calculated by taking the ratio of SA-β-gal-positive cells to the total cell count and multiplying the result by 100.

### 4.11. Recording of Whole-Cell Current 

Whole-cell current measurements were taken using an Axopatch 200 amplifier (Axon Instruments, Union City, CA, USA). The membrane potential was held at −80 mV, followed by a 1 sec depolarizing voltage pulse, varying between −120 and +60 mV. When filled with the pipette solution, the patch pipettes exhibited a resistance ranging from 4 to 5 MΩ. The pipette solution comprised (in mM) 150 KCl, 1 MgCl_2_, 5 EGTA, and 10 HEPES. The bath solution contained (in mM) 135 NaCl, 5 KCl, 1 CaCl_2_, 1 MgCl_2_, 5 glucose, and 10 HEPES. We used an isotonic solution (mM) containing 62 NaCl, 5 KCl, 1 CaCl_2_, 1 MgCl_2_, 5 glucose, 10 HEPES, and 150 mannitol to assess the currents changed by swelling. We reduced the mannitol to 50 mM in the same components for the hypotonic solution to lower the osmolarity to 200 mOsm. To achieve a pH of 7.3, adjustments were made using either HCl or NaOH (KOH). After the capacitative transients were canceled, whole-cell currents were measured. The pCLAMP software (Version 8, Axon) was utilized for whole-cell current analysis. Recordings were conducted under ambient conditions. 

### 4.12. Measurement of Cell Volume

Changes in the volumes of individual cells were determined by monitoring shifts in the concentration of a trapped fluorescent dye inside the cell, as documented in prior research [59]. CHO cells, which had been transfected with DNA that encodes rat TASK-3 (GenBank ID, AF192366) within the pcDNA3.1 vector, were cultured on 25 mm round coverslips. These cells were then treated with calcein AM at a concentration of 5 μM for 5 min. Before initiating the experiment, they were exposed to an isotonic solution for 30 min. All the experimental observations were made using a confocal laser imaging device (Olympus). The light source for excitation was set at 488 nm, and emitted light was detected at wavelengths exceeding 515 nm. The results are depicted using the F_0_/F_t_ notation. In this context, F and t denote fluorescence intensity and time, respectively. The value F_0_ indicates the fluorescence intensity when the cell is in an isotonic solution and the time is zero. The ratio F_0_/F_t_ serves as an indicator of the cell volume. 

### 4.13. Gene Silencing with Small Interfering RNA

Gene silencing assay was conducted according to established protocols [22]. GCs were transfected using either a scrambled siRNA as a negative control (NC, ON-TARGET Non-targeting Pool; Dharmacon, Lafayette, CO, USA) or TASK-3-specific ON-TARGETplus SMARTpool siRNA (Dharmacon). Transfections were performed in a serum-free medium using the Magnetofection™ system (Chemcell GmbH, Berlin, Germany) following the manufacturer’s guidelines. For the transfection mixture, 75 nM of either NC siRNA or rat TASK-3 siRNA was combined with 1.0 μL of PolyMAG (Chemicell GmbH, Berlin, Germany). The mixture was incubated for 20 min at room temperature before being added to 500 μL of serum-free culture medium in individual wells of a 24-well plate. The plate was then placed on a MagnetoFACTOR plate 24 device and incubated for 30 min at room temperature. Following this, the culture medium was replaced with fresh serum-containing medium and the cells were incubated for an additional 6 h at 37 °C in 95% air and 5% CO_2_. Subsequently, the medium was refreshed and the cells were allowed to grow for an additional two days. 

### 4.14. Data Analysis and Statistics

Images of agarose gels and Western blots were captured using a LAS-4000 luminescent image analyzer (Fujifilm Corp, Tokyo, Japan). Band intensities from gels and blotting membrane were quantified using Sigma Gel image analysis software (version 1.0, Jandel Scientific, San Rafael, CA, USA) or Quantity One software (version 4.6.3), which is compatible with a GS-800 calibrated densitometer (Bio-Rad, CA, USA). Data are presented as mean ± SD. Statistical significance was determined using Student’s *t*-test, with *p* < 0.05 considered significant. All statistical analyses were conducted using OriginPro2020 software (OriginLab Corp., Northampton, MA, USA).

## 5. Conclusions

This is the first study indicating a reduced TASK-3 channel gene in GCs from bovine cystic follicles. GCs with diminished TASK-3 expression displayed cellular senescence, contributing to cyst formation. Decreased TASK-3 expression disrupts water and K^+^ movement in GCs. TASK-3 may be vital in bovine follicular cyst mechanisms. Our findings offer insights into follicular cyst pathophysiology and contribute to building a genetic understanding of its onset. Furthermore, these results may help us understand human ovarian cysts because the female bovine reproductive tract shares many similarities with the human reproductive tract [60]. 

## Figures and Tables

**Table 1:** Primer sequences for PCR amplification of bovine samples.

Gene Name (Channel Name)	Species	GenBank Accession Number	Primer Sequences (5′–3′)	Application	Expected Size (bp)
*Kcnk3* (TASK-1)	Bovine	XM_597401	F: CAGGCCTACTACTACTGCT R: GGCCCGTGAGGATGTAGA	qRT-PCR	133
			F: ACACCTTCGTGAAGTACCTG R: GGATGTAGACGAAGCTGAAG	RT-PCR	287
*Kcnk9* (TASK-3)	Bovine	XM_588194	F: CTACTACTGCTTCATCACGTTG R: CCCACCAGGATATACATAAAGCTA	qRT-PCR	123
			F: CTACGTGGCCTTTAGCTTTA R: GTCGGTAAAGCTGTGTAACC	RT-PCR	433
*GAPDH*	Bovine	NM_001034034	F: ATGGTCTACATGTTCCAG R: AAGATGGTGATGGCCTTT	qRT-PCR	104
			F: CAGCGACACTCACTCTTCTAC R: GGAAGTCAGGAGATTCTCAGT	RT-PCR	250

## Data Availability

The data related to this study are not currently stored in a publicly accessible repository. However, the authors are willing to provide the data upon reasonable request. Requests for access to the data can be directed to Dawon Kang (dawon@gnu.ac.kr).
